# Indiana

**Published:** 1897-03

**Authors:** 


*Indiana.* Dr. W. W. Hamilton, of the State Board of Agriculture, recommends the abolishment of the Live-stock Sanitary Commission. This suggestion should receive the prompt attention of the people of Indiana, for it would be much more honorable to cut its head off at once than to allow the commission to die a slow death of starvation for want of money to work with.

